# Assessing the impact of aging and blood pressure on dermal microvasculature by reactive hyperemia optical coherence tomography angiography

**DOI:** 10.1038/s41598-021-92712-z

**Published:** 2021-06-28

**Authors:** Michael Wang-Evers, Malte J. Casper, Joshua Glahn, Tuanlian Luo, Abigail E. Doyle, Daniel Karasik, Anne C. Kim, Weeranut Phothong, Neera R. Nathan, Tammy Heesakker, Garuna Kositratna, Dieter Manstein

**Affiliations:** 1 Cutaneous Biology Research Center, Massachusetts General Hospital, Harvard Medical School, Boston, MA USA; 2 Laboratory for Functional Optical Imaging, Department of Biomedical Engineering, Columbia University, New York, NY USA; 3 Yale School of Medicine, New Haven, CT USA; 4 Department of Dermatology, Siriraj Hospital, Mahidol University, BKK, Thailand

**Keywords:** Translational research, Medical imaging, Cardiovascular biology, Skin manifestations

## Abstract

Visualization and quantification of the skin microvasculature are important for studying the health of the human microcirculation. We correlated structural and pathophysiological changes of the dermal capillary-level microvasculature with age and blood pressure by using the reactive hyperemia optical coherence tomography angiography (RH-OCT-A) technique and evaluated both conventional OCT-A and the RH-OCT-A method as non-invasive imaging alternatives to histopathology. This observational pilot study acquired OCT-A and RH-OCT-A images of the dermal microvasculature of 13 young and 12 old healthy Caucasian female subjects. Two skin biopsies were collected per subject for histological analysis. The dermal microvasculature in OCT-A, RH-OCT-A, and histological images were automatically quantified and significant indications of vessel rarefaction in both old subjects and subjects with high blood pressure were observed by RH-OCT-A and histopathology. We showed that an increase in dermal microvasculature perfusion in response to reactive hyperemia was significantly lower in high blood pressure subjects compared to normal blood pressure subjects (117% vs. 229%). These results demonstrate that RH-OCT-A imaging holds functional information of the microvasculature with respect to physiological factors such as age and blood pressure that may help to monitor early disease progression and assess overall vascular health. Additionally, our results suggest that RH-OCT-A images may serve as a non-invasive alternative to histopathology for vascular analysis.

## Introduction

Aging is a complex process that can be characterized by decreased maximal function, lower reserve capacity, and the diminishing ability of systems within the body to perform normal functions in day-to-day life^1^. In addition to intrinsic aging which primarily causes functional changes^2^, environmental factors such as sun exposure can affect both the appearance and function of dermal microvasculature^3^. In specific for the population of 65 years and older, there is a significant increase in intrinsic vascular aging and blood pressure which results in stiffening of the vascular walls and vessel rarefaction, and in particular, loss of the capillary loops that occupy the dermal papillae^4–7^. Therefore, we hypothesized that older individuals with higher blood pressure would have a reduced dermal vascular density and reserve capacity, and that investigating both dermal microvascular structure and function could provide important insights into underlying pathophysiological conditions.

Despite being easily accessible, it is technically challenging to image and quantify the function and structure of the dermal microvasculature in vivo to study the health and disease of the human microcirculation. While current techniques for in vivo dermal microvasculature imaging, such as capillaroscopy, intravital microscopy, and laser doppler imaging, are limited to specific regions (nail fold and extremities), require exogenous contrast agents, or have poor spatial resolution^8–10^, optical coherence tomography angiography (OCT-A) is a label-free imaging technique that overcomes these limitations and captures the baseline perfusion of capillary-level microvasculature^11,12^. OCT-A devices employ multiple scans of low-coherence light onto the same tissue cross-section. Variations in the backscattered signal between each sequential scan are caused by moving blood cells, thereby denoting areas in which there is blood flow. Scans of several adjacent cross-sections are then combined to construct 3D perfusion maps^13^.

Conventional OCT-A, however, only captures currently perfused vessel structures, which provides an incomplete illustration of the skin microvasculature given that the dermal perfusion constantly changes to maintain the body’s homeostasis^14,15^. Recently, it has been demonstrated that a reactive hyperemia related increase in blood perfusion during the OCT-A image acquisition can enhance the visualization and quantification of the microvascular network^16–19^.

Reactive hyperemia is characterized by increased blood flow following a brief period of vascular occlusion. The purpose of this regionally increased blood flow is to quickly remove metabolic wastes and provide oxygen to ischemic tissues for the prevention of ischemic damages. Reactive hyperemia is a well-documented peripheral microvascular function which has been linked to clinical conditions such as endothelial dysfunction and cardiovascular health^20–22^. Measurement of reactive hyperemia is typically performed by placing and inflating a pneumatic pressure cuff on the upper arm to occlude the forearm blood flow for 1–5 min ^20,23^. After the occlusion is released, the reactive hyperemia response can be measured by several techniques such as laser Doppler flowmetry (LDF) and peripheral artery tonometry (PAT)^24,25^.

**Figure 1:**
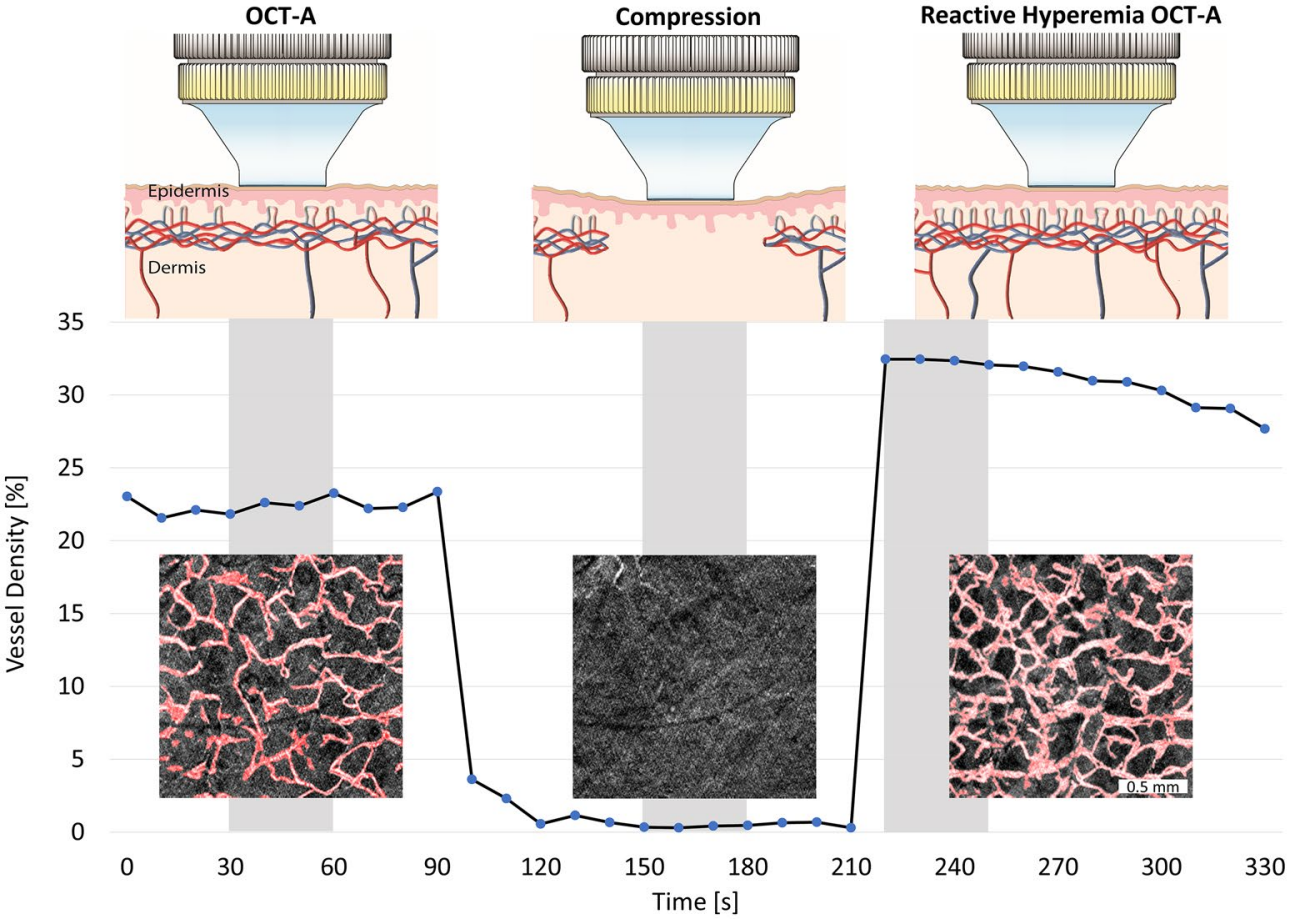
Scheme of the image acquisition protocol and microvascular perfusion response to the application and subsequent release of mechanical stress. For this graph, OCT-A images (1.75 mm × 1.75 mm) of the forearm of a representative young subject were acquired every 10 s. For the rest of the study population, 3 OCT-A images (6 mm × 6 mm) were acquired during the highlighted gray areas. Each image acquisition took 30 s followed by 30 s of data processing and saving. After the baseline OCT-A image is captured, mechanical stress (20–90 kPa) is induced for 2 min. In the meantime, the compression state image is acquired. Immediately after releasing the compression, the reactive hyperemia OCT-A image is taken. OCT-A images are shown with the resulting automatic vessel segmentation map overlaid in red, which also constitutes the vessel density measure.

For this study, we applied mechanical stress to the forearm to induce a reactive hyperemia response as it has been shown to be equally or even more effective compared to cuff occlusion^26–28^. After release of the applied pressure, the reactive hyperemia response temporarily allows one to capture OCT-A images at increased perfusion (Fig. 1) before the dermal blood flow returns to resting value. Therefore, reactive hyperemia OCT-A (RH-OCT-A) provides additional visual information compared to LDF and PAT and illustrates the skin microvasculature at increased perfusion compared to baseline perfusion demonstrated using conventional OCT-A. The RH-OCT-A images of the vascular network at increased perfusion also allow for an improved comparison with histopathology.

Histopathological approaches are the current gold standard for morphological vessel analysis, which, independently of perfusion, visualize the entire vascular network. Although histopathology is commonly used in microvasculature research and in clinics to characterize a variety of pathophysiological characteristics, it is labor-intensive and involves both suture care and risk of infection^29^. RH-OCT-A, on the other hand, provides fast, label-free, and non-invasive acquisition of high-resolution vasculature images in vivo that reflect biological conditions and biomechanical features. These defining features of RH-OCT-A provide additional advantages over other commonly used perfusion measurements such as laser doppler perfusion imaging and laser speckle contrast imaging^10,18,19^.

Furthermore, we quantified the functional status of the forearm microvasculature by comparing dermal baseline perfusion of conventional OCT-A images to peak reactive hyperemia perfusion of RH-OCT-A images. We propose that this comparison could be used to provide a more functional analysis of the microvasculature similar to the flow-mediated dilation and the reactive hyperemia peripheral artery tonometry techniques which are used to measure endothelial function of vessels^30^.

These advantages of RH-OCT-A imaging enable clinicians to dynamically visualize and characterize the microvasculature of each patient over time. New features and improved quantifications discussed in this work might help to broaden the clinical utility of OCT systems which are often limited by the system cost, image acquisition usability and practicality, as well as data interpretation^12,31^. In this respect, our study both seeks to show that the quantitative analysis of RH-OCT-A images provides a functional characterization of the dermal microvasculature and establishes RH-OCT-A as a non-invasive clinical research alternative to histopathology for vascular analysis.

## Results

### Quantitative analysis of OCT-A and RH-OCT-A images

**Figure 2:**
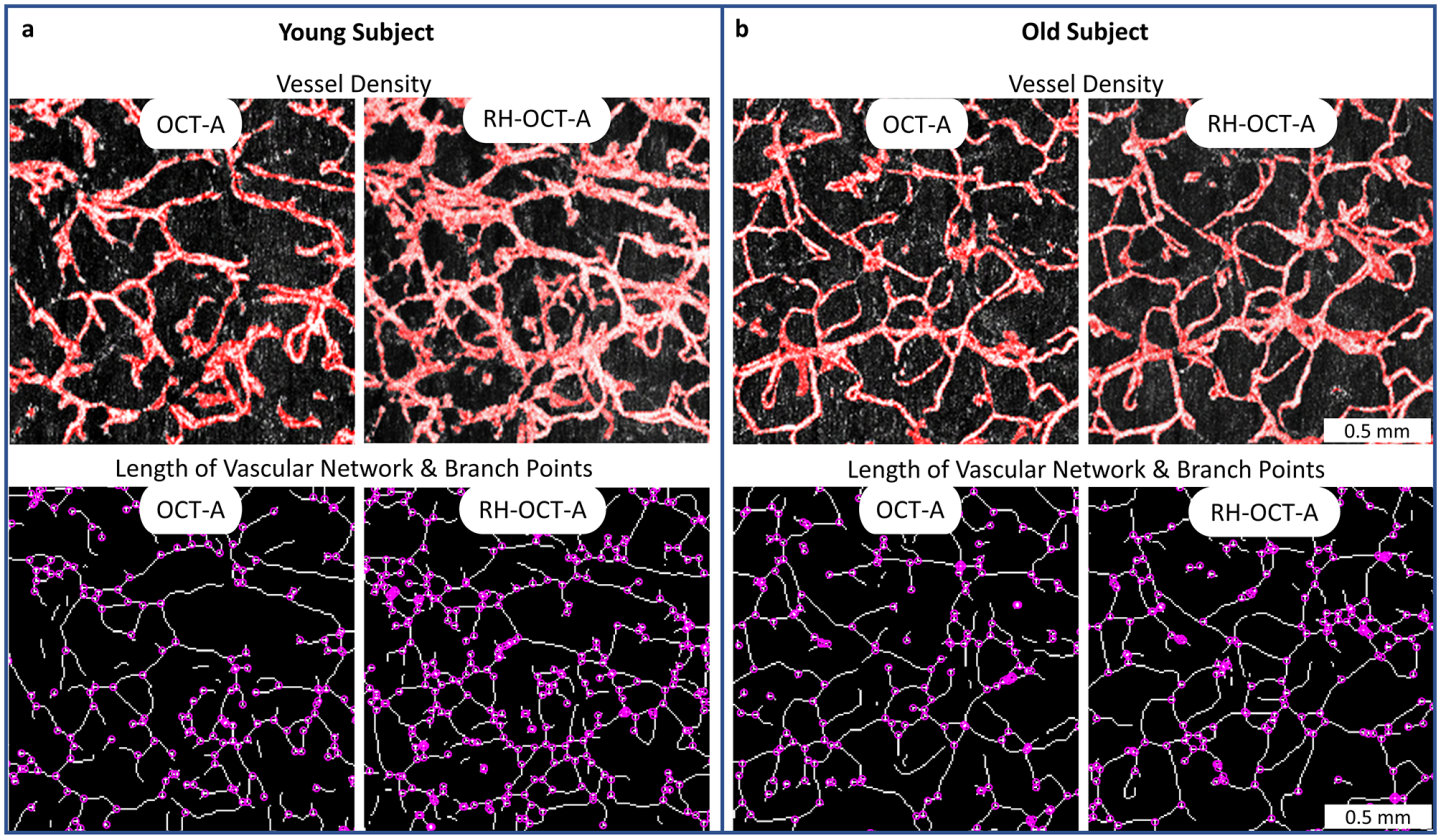
Imaging of human dermal microvasculature. (**a**) Baseline perfusion (OCT-A) images and reactive hyperemia (RH-OCT-A) images showing the vessel density (VD, red colored overlay) of a representative young (age: 25 years; systolic blood pressure: 117 mmHg) and (**b**) old subject (age: 66 years; systolic blood pressure: 136 mmHg). The skeleton maps of the vessel network (1-pixel trace width) show the length of the vascular network (LVN, white pixel) and number of vessel branch points (NBP, purple dots).

OCT-A and RH-OCT-A images were acquired from 13 young and 12 old subjects at the same location of the inner and outer forearm. Classical OCT-A measurements (Fig. 2) captured the baseline perfusion of the microvascular network. The microvasculature was automatically segmented and analyzed by quantifying the vessel density (VD), length of the vascular network (LVN), and number of branch points (NBP)^32^. At the peak of reactive hyperemia perfusion (Figs. 1, 2), vessels of the dermal network appear more detailed and prominent, and new vessels and branch points emerge resulting in significantly increased VD, LVN, and NBP, compared to classical OCT-A baseline perfusion images (Table 1). Most vessels visible in the baseline image increased in diameter in the reactive hyperemia image. Yet, the overall average vessel diameter (diameter = VD/LVN) decreased from baseline to peak reactive hyperemia perfusion images for young (31.7–30.4 µm, respectively) and old subjects (29.7–28.6 µm, respectively) due to the newly emerging and comparatively thinner vessels (Table 1).

**Table 1:** Impact of age and systolic blood pressure (SBP) on the vasculature assessed by optical coherence tomography angiography (OCT-A), reactive hyperemia optical coherence tomography angiography (RH-OCT-A), and histology.

Method	Cohort	Vessel density (%)	Length of vascular network (mm)	Number of branch points	Vessel diameter (µm)
OCT-A	Young	22.7 (±2.0)	7.2 (±0.6)	2275 (±320)	31.7 (±2.7)
	Old	21.2 (±2.5)	7.1 (±0.8)	2320 (±370)	29.7 (±2.0)
	SBP < 130	21.9 (±2.5)	7.1 (±0.8)	2250 (±330)	30.8 (±2.7)
	SBP ≥ 130	21.9 (±2.8)	7.2 (±0.8)	2330 (±340)	30.6 (±2.6)
RH -OCT-A	Young	30.7 (±1.7)	10.2 (±0.8)	3730 (±440)	30.4 (±1.9)
	Old	26.9 (±3.3)	9.4 (±1.4)	3245 (±660)	28.6 (±2.0)
	SBP < 130	30.0 (±2.6)	10.2 (±0.8)	3710 (±460)	29.6 (±2.2)
	SBP ≥ 130	26.9 (±3.1)	9.2 (±1.2)	3095 (±575)	29.5 (±2.2)
Histology	Young	27.4 (±4.8)	12.3 (±1.7)	5895 (±1025)	22.3 (±2.6)
	Old	24.3 (±5.4)	11.1 (±2.0)	5065 (±1205)	22.0 (±2.5)
	SBP < 130	27.6 (±4.7)	12.3 (±1.7)	5940 (±1075)	22.3 (±2.3)
	SBP ≥ 130	23.2 (±5.3)	10.6 (±1.7)	4838 (±1070)	21.8 (±2.9)

### Effects of age on dermal microvasculature

**Figure 3:**
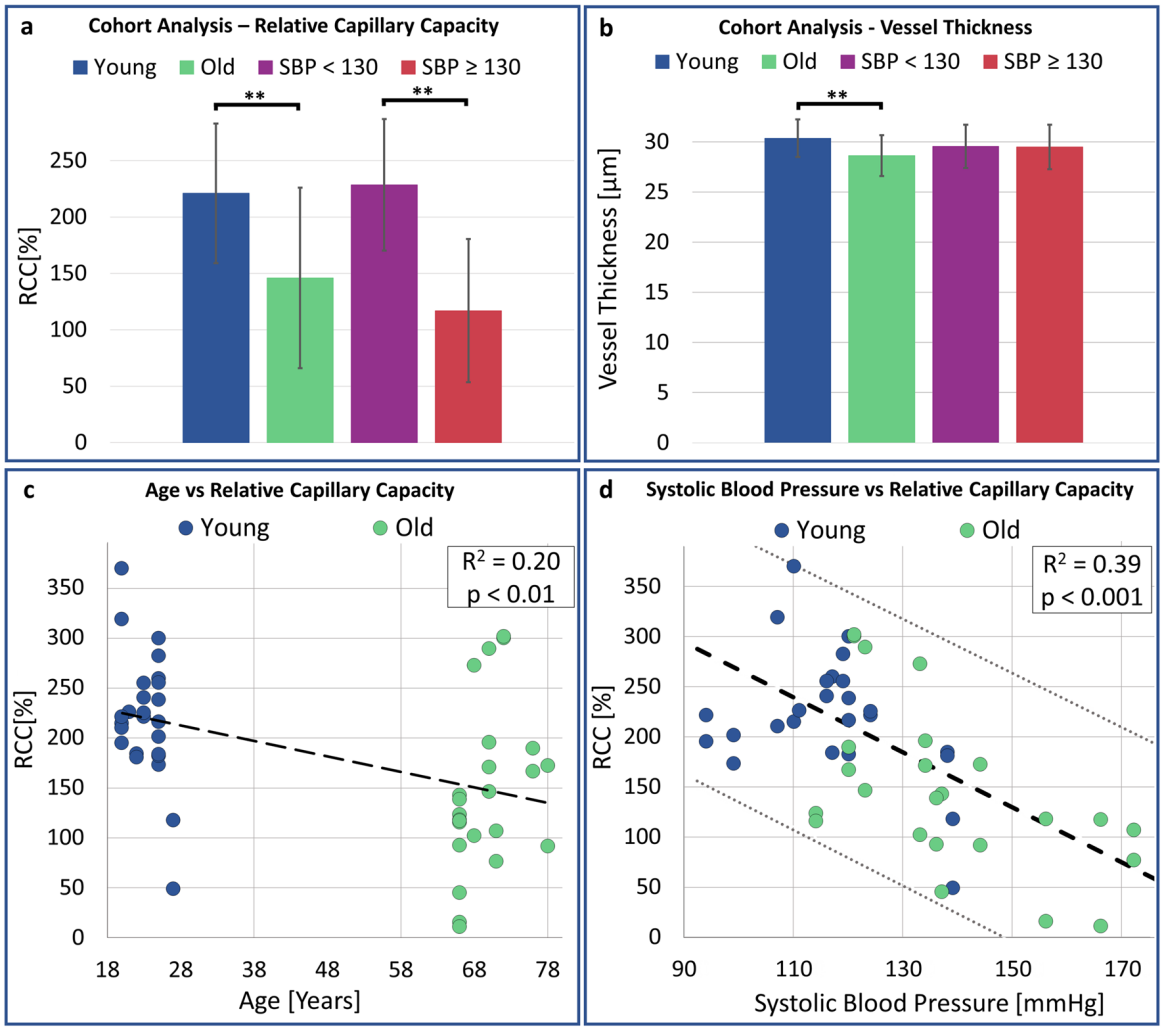
Impact of age and systolic blood pressure on the skin’s perfusion relative capillary capacity and vessel diameter. (**a**) The cohort analysis shows significant differences in RCC values and (**b**) vessel diameter in relation to age and systolic blood pressure. 2 RH-OCT-A images per subject (young: n = 13; old: n = 12; SBP < 130: n = 15; SBP ≥ 130: n = 10). (**c**) The scatterplot shows a significant correlation between age and the RCC metric (*R*^2^ = 0.2, *p* < 0.01) and (**d**) systolic blood pressure and RCC values (*R*^2^ = 0.39, *p* < 0.001). *R*^2^ is the square of the Pearson correlation coefficient. 2 RH-OCT-A images (inner and outer forearm) were obtained per subject (n = 25).

In Table 1, OCT-A baseline images did not show significant differences in microvascular perfusion between age groups as reflected in each of the metrics: mean VD (22.7% vs. 21.2%), LVN (7.2 mm vs. 7.2 mm), and NBP (2275 vs. 2320). Upon peak reactive hyperemia perfusion (RH-OCT-A), both age groups showed a significant increase within each metric. Comparing conventional OCT-A and RH-OCT-A images by combining their quantitative values (VD, LVN, and NBP) into a single metric, namely, the relative capillary capacity (RCC, Methods Eq. 1) allowed for an indicative value to quantify functional vascular changes. A significant correlation between age and the RCC metric was observed (*R*^2^ = 0.2, *p* < 0.01, Fig. 3c). Young subjects had greater vasculature perfusion potential compared to old subjects, as shown by the difference in RCC values (221% vs. 146%, respectively) (Fig. 3a). Surprisingly, no significant changes between ventral and dorsal forearm microvasculature were found among the young and old groups (Table S1). Therefore, the ventral and dorsal images were combined, with 26 and 24 data points in each respective age group. As expected, the vessel diameter significantly decreased with age for both the OCT-A and RH-OCT-A images (Table 1; Fig. 3b).

### Effects of blood pressure on dermal microvasculature

We found the strongest correlation (*R*^2^ = 0.39, *p* < 0.001) between the RCC metric and systolic arterial pressure (Fig. 3d) when comparing quantitative microvascular metrics with clinical metrics. 15 subjects had a resting systolic blood pressure below 130 mmHg (11 young, 4 old) and 10 subjects had a resting systolic blood pressure above 130 mmHg (2 young, 8 old). While no significant differences in baseline perfusion (OCT-A) derived metrics can be drawn between the normal and high blood pressure groups (VD: 21.9% vs. 21.9%; LVN: 7.1 mm vs. 7.2 mm; NBP: 2250 vs. 2330), there were significant differences (*p* < 0.01) for the RH-OCT-A images (VD: 30.0% vs. 26.9%; LVN: 10.2 mm vs. 9.2 mm; NBP: 3710 vs. 3095) as seen in Table 1. The RCC values increased up to 229% in subjects with normal blood pressure, while only a 117% increase was seen in subjects with high blood pressure (Table 1; Fig. 3b, *p* < 0.001). Interestingly, we found no significant correlation between diastolic blood pressure and the RCC metric (*R*^2^ = 0.07). In addition, the vessel diameter of OCT-A and RH-OCT-A images did not change significantly for subjects with normal (30.8–29.6 µm, respectively) and high systolic blood pressure (30.6–29.5 µm, respectively).

### Vessel histology compared to RH-OCT-A images

**Figure 4:**
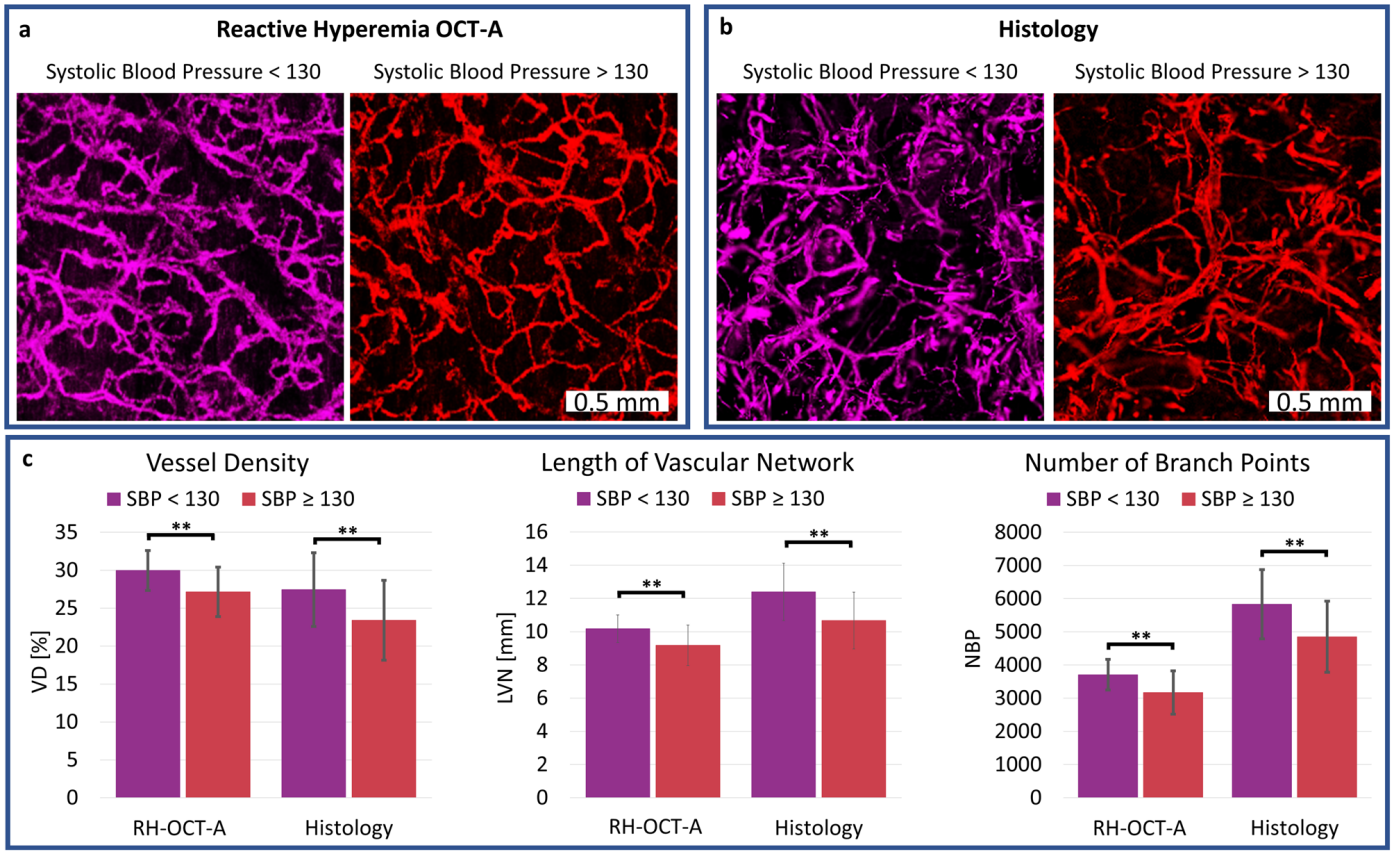
Comparison of RH-OCT-A images and equivalent horizontal histological images, both representing the microvasculature from a depth range of 40–440 µm of the upper dermis. Shown are two representative (**a**) RH-OCT-A images and (**b**) histology images: colored in purple is a subject with normal blood pressure and colored in red is a subject with high blood pressure. (**c**) Quantification of vessel density (VD), length of the vascular network (LVN), and number of branch points (NBP) for normal and high blood pressure subjects of RH-OCT-A images and histology images. **p < 0.01. Values represent the mean value and the standard deviation of the mean from 2 RH-OCT-A images and 2 histological images obtained for each subject (SBP < 130: n = 15; SBP ≥ 130: n = 10).

Horizontal histology images were compared to RH-OCT-A images, both representing the microvasculature from a depth range of 40–440 µm (Fig. 4a, b). In both the histology and RH-OCT-A images (*p* < 0.01), subjects with high blood pressure had significantly reduced vessel density, length of the vascular network, and number of branch points compared to subjects with normal blood pressure levels (Fig. 4c; Table 1). Although histological and RH-OCT-A images were acquired at the same location, clear morphological differences among the images were observed. These differences can be attributed to known characteristics of the biopsy and histology process such as tissue and vessel shrinkage as well as inaccurate alignment of the horizontal histological sections relative to each other^33^. The fact that the reactive hyperemia response increases blood perfusion but does not necessarily perfuse the entire dermal microvasculature can also attribute to structural differences between RH-OCT-A images compared to histology. Another contributing factor to these differences could be our use of a local anesthetic that contains epinephrine which has the potential to cause histological vasoconstriction^34^. Vascular network images acquired via histology showed poor connections between layers (Fig. 4a, b) and a considerably higher number of branch points and length of the vascular network, as well as a lower vessel density compared to RH-OCT-A images (Fig. 4c; Table 1). Nevertheless, both histology and RH-OCT-A confirm a significant correlation between vessel measurements among the study groups (Fig. 4c). However, we did not find that such a correlation exists for pairwise RH-OCT-A measurements and histology measurements of individual subjects (Fig. S1). We compared the microvasculature of each subject’s inner and outer forearm and observed no correlation (*R*^2^ = 0.09, *p* = 0.15) through histological evaluation and found a significant correlation (*R*^2^ = 0.52, *p* < 0.001) for the RH-OCT-A images (Fig. S2). The RH-OCT-A results demonstrated that no significant microvasculature differences are noticeable between the inner and outer forearm. These results illustrate the consistency of the RH-OCT-A method and highlight some of the tissue processing related drawbacks of histopathology in vascular analysis, especially when analyzing horizontal sections.

## Discussion

In this study, we describe the use of conventional OCT-A, RH-OCT-A, and histological quantification of the dermal microvasculature on female adults from 2 different age groups to highlight the potential role of these diagnostic techniques in detecting both structural and functional changes to the microvasculature. Given that a recent OCT-A study investigating facial microvasculature demonstrated differences in vessel density between age groups, we initially expected to find differences in baseline perfusion of the skin amongst young and old study groups as well^35^. However, here, the forearm showed no differences in baseline perfusion of OCT-A images between the age groups, likely due to regional microvasculature differences between the face and arm. Instead, we found a higher vasculature density and far greater RCC values in young subjects compared to old subjects by analyzing the RH-OCT-A data. Interestingly, blood pressure, rather than age, was the key microvascular feature that impacted this finding.

We report several findings that are important in advancing our understanding of microvascular changes at the dermal capillary level with respect to blood pressure. According to the American Heart Association, cases of high systolic blood pressure and hypertension are linked to consistent readings over 130 mmHg^36^. Out of the 13 young subjects, 11 had a normal systolic blood pressure (< 130 mmHg) and 8 of the 12 old subjects had a “high” systolic blood pressure (≥ 130 mmHg).

The microvasculature quantification in our study revealed that subjects with high systolic blood pressure had a lower dermal vasculature density compared to subjects with normal systolic blood pressure (Table 1). This was expected since hypertension is known to cause stiffening and rarefication of vessels, due to vessel constriction and apoptosis of endothelial cells^6,37–39^. Whether rarefaction is a cause or consequence of hypertension remains unclear^4,37^, however, it has been suggested that it may precede the elevated blood pressure^39,40^. Therefore, the use of RH-OCT-A may be the adequate research tool to diagnose and study disease processes and interventions that influence vascular health.

While our results seem to suggest that an increase in blood pressure, rather than in age, is the culprit in functional dermal microvasculature changes, it is well known that age is a significant risk factor for high blood pressure^36^, and thus, the two remain linked. Surprisingly, only old study subjects and not those with high blood pressure showed a significant reduction in vessel diameter as determined by OCT-A and RH-OCT-A. The mechanisms underlying the relation between age, systemic blood pressure, and capillary rarefaction are unclear based on the present data. However, it may be that subjects with high systemic blood pressure are more prone to develop chronic hypertension and arteriosclerosis over time, where vessels become narrower with age, leading to increased resistance to blood flow^41^. Therefore, future studies should focus on subjects with suspected coronary artery disease and compare OCT-A derived metrics with common biomarkers such as low‐density lipoprotein cholesterol, total cholesterol, and C‐reactive protein^42^.

While previous studies^43,44^ have shown microvascular dilation after short-term UV exposure, we did not find significant differences between the outer (sun-exposed) and inner (less sun-exposed) forearm using OCT-A, RH-OCT-A, and histopathology (Table S1). Our findings suggest that either the dermal microvasculature is not chronically affected, or that the difference of UV induced damage between the outer and inner forearm was not sufficient. While further studies are warranted to analyze the microvascular impact of chronic UV exposure, less sun-exposed body regions should be examined for the assessment of pathophysiological changes such as intrinsic ageing and blood pressure^3^.

Another goal of this study was to compare the RH-OCT-A method to histopathology. As hypothesized, both RH-OCT-A and histopathology demonstrated a significant correlation of microvasculature rarefaction in old subjects and subjects with high blood pressure as well as high consistency of all quantitative vessel metrics (Table 1; Fig. 4). However, the inability to pairwise correlate individual samples between the two analysis methods could be due to histological distortion and artifacts as well as inadequate occlusion and reperfusion of the entire microvasculature during RH-OCT-A imaging. This inconsistency highlights histopathological imprecisions^33,45^ when analyzing microvasculature where vessel specimens are known to both collapse and dehydrate^33,35,45^. It also shows limitations of RH-OCT-A imaging which need to be investigated in further studies. These methodological differences could explain why in vivo imaging (OCT-A and RH-OCT-A), and not ex vivo histopathology, detected significant age-related changes in vascular diameter which could arise from physiological phenomena such as age-related chronic vasoconstriction and impaired endothelium-dependent vasodilation of the microvasculature^7^.

Future studies should consider the in vivo assessment of vessel diameter by OCT-A and RH-OCT-A to improve the diagnosis of cutaneous vasculitis, an inflammation of the small blood vessels of the skin, which is classified by the size of vessels affected^46^. In addition, histopathology is limited to only providing morphological information, whereas OCT-A and RH-OCT-A have been shown to provide functional microvasculature information in response to local stimuli such as topical drugs, mechanical distortion, and temperature changes^16,47^. As such, we conclude that for dermal microvasculature visualization, RH-OCT-A has the potential to serve as a non-invasive alternative for histopathology^35,45^.

To analyze physiological decrements that are the result of aging and increased blood pressure, we determined the skin’s peak reactive hyperemia perfusion, vessel diameter, and RCC values. Our results show that these values are connected to a subject’s current performance potential which is related to physiological factors and vascular health. Though additional studies are needed to correlate the link between reactive hyperemia perfusion of the microvasculature with vascular health, RH-OCT-A imaging shows promise since functional and time course measurements lack visual information or are not feasibly obtained using traditional approaches^24,48^.

Furthermore, considering ocular OCT-A studies that discuss various disease correlated peripapillary microvasculature changes^49,50^, the RH-OCT-A method could become indispensable as a prognostic tool for a variety of clinical conditions with vascular manifestations, such as diabetes, arteriosclerosis, Buerger's disease, chronic venous insufficiency, and systemic sclerosis^8,51–54^. These conditions are characterized by early impairment of the microvasculature, along with progressive tissue fibrosis, which can cause inadequate blood circulation, tissue damage, and even capillary infarction^8,55^. Since microcirculatory abnormalities have been demonstrated to precede clinical symptoms^56^, RH-OCT-A screening may help with early detection and implementation of preventative measures to mitigate or resolve the underlying pathology before the onset of permanent damage. Like capillaroscopy, high frequency ultrasound, and laser doppler imaging, the RH-OCT-A method could potentially be used to make a differential diagnosis between primary and secondary Raynaud's phenomenon, which usually presents in the fingers or toes, more rarely in the nose and ears, and involves a sequence of skin color changes, attributed to vasospasm and subsequent vessel dilation^57^.

We envision applying RH-OCT-A and its derived metrics such as the vessel density and RCC values for close monitoring and clinical diagnosis of small vessel occlusive diseases like systemic lupus erythromatosis, mixed connective tissue disease, scleroderma, superficial thrombophlebitis, and conditions with ischemic manifestations as is commonly seen in the extremities of diabetic patients and life-long smokers^58^. RH-OCT-A could also be specifically used to probe for signs of peripheral artery disease in the legs or lower extremities in which narrowed blood vessels outside the heart cannot deliver enough oxygen and nutrients to the body. While it is not life threatening, it can affect one’s quality of life, and can be a warning sign for more serious conditions, including heart disease^59^. Similar to the ankle-brachial index test which compares the blood pressure measured at the ankle with the blood pressure measured at the arm, clinicians could potentially use RH-OCT-A to diagnose peripheral artery disease from the onset by looking for vasculature and cutaneous manifestations.

Although the specific research equipment used in this study has yet to become obtainable in clinics, other cart-based dermatologic OCT and OCT-A systems with handheld probes are commercially available, easily transportable across different clinical settings, and FDA approved^60,61^. In addition, OCT-A equipment is readily accessible and only minor software and hardware updates to these devices are needed to obtain similar results to the ones presented in this paper.

The results of this study are significant and informative; however, the findings must be considered from a clinical perspective and in light of the study design. A limitation of our study is the relatively small sample size and therefore, the results and the predictive value of the RCC metric need confirmation in a larger patient population. In addition, the lengthy RH-OCT-A image acquisition prevents the observation of age-related changes in reactive hyperemia response times^26^. With regards to the study design, another limitation is that only a single blood pressure measurement per subject was acquired. Future research should assess the subject’s blood pressure history. Another consideration for our study is that though we selected a homogenous study population (health status, gender, race, age), various factors known to affect the microvasculature and endothelial function have not been investigated such as homocysteine levels, advanced glycation end products, cholesterol levels, glucose levels, triglyceride levels, hydration status, and physical activity^62–65^. Nevertheless, the statistical results of this study are supported by taking these factors into consideration, suggesting that there is a correlation between microvascular function and both age and systolic blood pressure. Finally, to better control the applied pressure, future studies should automate the procedure to enhance ease of use and make it independent of the clinician.

Our pilot study has demonstrated the superior clinical utility of RH-OCT-A imaging to analyze the dermal microvasculature as it quantifies dermal perfusion at the peak of reactive hyperemia which can highlight significant pathological differences where conventional OCT-A fails to provide enough information. We have also shown that the RH-OCT-A method may be used as an alternative to histopathology in order to non-invasively analyze the dermal microvasculature. In addition to the structural information provided by histopathology, OCT-A and RH-OCT-A methodologies have shown that the dermal capillary density and vessel diameter decrease with age and that some of the microvascular functions significantly decline with increasing age and blood pressure. These findings establish the framework for a more holistic and functional microvasculature assessment and encourage future research to elucidate the clinical significance and utility of these optical and non-invasive analysis methods.

## Material and methods

### Study design and subjects

To account for known microvascular differences among race and gender groups^66,67^, and to control for melanin absorbance of the OCT light^68^, healthy female Caucasian (Fitzpatrick skin type I–III) subjects with no history of smoking were recruited (Table S2). To emphasize aging effects and to minimize the number of study participants, a group of 13 young (18–30 years old) and 12 old (≥ 65 years old) subjects were enrolled rather than having an even distribution covering all ages. Subjects with reported underlying clinical conditions that required systemic medication in the past 6 months were excluded from the study. OCT-A imaging sequences and 4 mm full thickness skin punch biopsies were obtained from the non-dominant inner and outer forearm of each subject. Prior to biopsy acquisition, a single blood pressure measurement was taken while the subject relaxed in a sitting position in a temperature-controlled room for a minimum of 30 min. The anesthesia consisted of a local injection of lidocaine HCl 1% and epinephrine 1:100,000.

### OCT-A and RH-OCT-A acquisition

An OCT scanner (TEL220C1, Thorlabs) with a central wavelength of 1300 nm was used to acquire angiographic volumes with an axial resolution of 4.2 µm, a lateral resolution of 13 µm (LSM03, Thorlabs), and a dimension of 6 × 6 × 1.5 mm^3^ (spatial FOV: 924 × 924 × 430 voxel; Nyquist-sampled spacing: 6.5 × 6.5 × 3.5 µm) at an A-scan rate of 76 kHz. The angiographic volumes were converted into two-dimensional top-down projection images, representing the microvasculature from a depth range of 40–440 µm. To ensure the microvasculature images are in focus (depth of focus ≈ 300 µm), the OCT beam focus was positioned at a depth of around 150 µm below the epidermis. Unlike most clinical OCT probes (ring shaped), for our study, the subject's forearm was positioned beneath a full contact glass OCT probe (IMM3, OCTG-1300, Thorlabs) and glycerol was applied for index matching and as an immersion fluid (Fig. S3). Using a full contact OCT-A probe allows for even pressure distribution to the skin as monitored by a force sensor (FlexiForce A401, Tekscan, Boston, MA, USA). Three sequential OCT-A volumes were acquired at the same location of the inner and outer forearm (6 volumes total) of each subject and each volume acquisition took approximately 30 s with an additional 30 s for saving the data. First, a conventional OCT-A measurement was performed to capture baseline perfusion of the microvasculature (Fig. 1). Next, the OCT probe was used to induce mechanical stress (20–90 kPa). Due to the wide inter subject range of mechanical stress needed to suppress blood flow and to increase ease of use, the subject’s skin was compressed until restriction of perfusion was apparent via no speckle movement on the OCT B-scan live viewer and visible blanching of the skin as seen on the live camera image. This compression was held for 2 min. For each subject a compressed state OCT-A image was captured. The purpose of the compression image was to confirm complete inhibition of blood flow. Immediately after the OCT probe was released from its 2-min-long compressing state, the reactive hyperemia OCT-A (RH-OCT-A) image was acquired. A micrometer scale was used to ensure that baseline and reactive hyperemia OCT-A images were acquired at the same position and since the baseline and RH-OCT-A images are acquired in full contact mode, the change of focus between the two images was minimal (Fig. S4).

### Histology and whole slide scanning

Two 4 mm skin biopsies were obtained from the inner and outer forearm of each subject and then stored overnight in 4% paraformaldehyde and transferred to 30% sucrose for 2 days. Tissue was embedded into optimum cutting temperature solution (O.C.T. compound, Tissue-Tek) and stored at − 80 °C until sectioning. 10 sequential horizontal dermal sections from a depth range of 40–440 µm were collected and vessels were stained with endothelial marker CD31 (ab28364, Abcam). Two-dimensional images were generated by co-registering volumetric data using the StackReg plugin (ImageJ, NIH)^69^ and by using a maximum intensity algorithm (Fig. S5). Horizontal histopathology, instead of classical vertical cross sections, allowed for the automatic segmentation of the microvasculature as well as direct comparison of the histological samples with corresponding OCT-A and RH-OCT-A images.

### Vessel segmentation and image analysis

Movement artifacts, which presented primarily as stripes along the fast-scanning axis of the acquired image, were eliminated using combined wavelet and Fourier filtering (Fig. S6)^70^. All data sets were deidentified and automatically segmented vessels (Fig. 2) were analyzed by various quantitative metrics described in detail in earlier works^17,32^. In summary, the vessel density (VD) is based on the area of segmented vessels (red pixels) as a percentage of the entire image (Fig. 2)^7^. The skeleton map (Fig. 2) which shows vessels with a width of 1 pixel (6.5 µm) was used to determine the length of the vascular network (LVN) per mm^2^. The number of branch points (NBP) (purple dots) indicates the number of junctions (Fig. 2). Comparing quantitative values of the skin’s baseline perfusion of conventional OCT-A and the peak hyperemia perfusion of RH-OCT-A allowed for the determination of the relative capillary capacity:

1 $${\text{Relative}}\,{\text{Capillary}}\,{\text{Capacity }} = \left( {\frac{{VD_{{peak}} }}{{VD_{{baseline}} }}*\frac{{LVN_{{peak}} }}{{LVN_{{baseline}} }}*\frac{{NBP_{{peak}} }}{{NBP_{{baseline}} }}} \right) - 1$$

The relative capillary capacity (RCC) metric is a ratio that is expressed as a percentage change and it should be seen as a statistical convenience that permits information to be collected from multiple variables. While the relative capillary capacity is not a physiological metric it could help as an indicative value to quantify dynamic vascular changes in response to capillary wall stiffness, vessel permeability, and other factors^17^. MATLAB code and example data are archived at https://github.com/MichaWangEvers/Vessel-Segmentation.

### Statistical analysis

*T*-tests were used to compare vessel density (VD), length of the vascular network (LVN), number of branch points (NBP), and RCC measurements between blood pressure groups (normal and high) and age groups (young and old). For correlation analysis, we used linear regression analysis to evaluate the association of age and systolic blood pressure with RCC values.

### IRB statement

All procedures were approved by the Institutional Review Board of the Massachusetts General Hospital (Protocol No.: 2018P001115) and informed consent was obtained from all subjects. All research methods were performed in accordance with relevant guidelines and regulations.

## Supplementary Information

Supplementary Informations.
