# Supplementary material for: Assessing the impact of aging and blood pressure on dermal microvasculature by reactive hyperemia optical coherence tomography angiography
Source: Sci Rep. 2021 Jun 28;11:13411. doi: 10.1038/s41598-021-92712-z (PMC8238964; doi:10.1038/s41598-021-92712-z)
Supplement: Supplementary file 1 — Supplementary Informations. [file 41598_2021_92712_MOESM1_ESM.docx]

Supporting Information

Assessing the impact of aging and blood pressure on dermal microvasculature by reactive hyperemia optical coherence tomography angiography


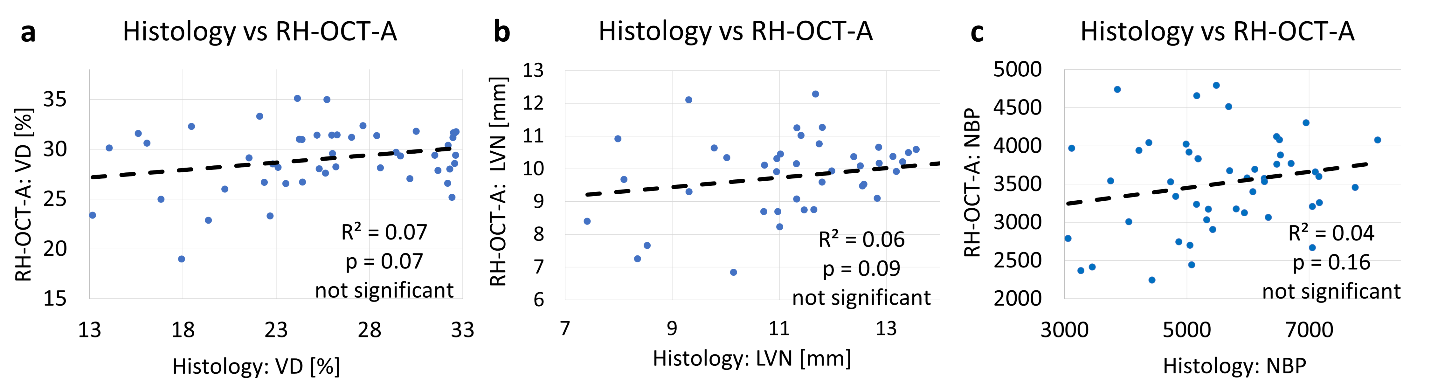


**Fig. S1.** Correlation between histology and RH-OCT-A images. There is no direct correlation for any microvascular metric (**a**) vessel density (VD), (**b**) length of the vascular network (LVN), and (**c**) number of branch points (NBP) when analyzing samples of individual subjects with RH-OCT-A and histology. This was most likely caused by histology artifacts such as shrinkage and dehydration. R^2^ is the square of the Pearson correlation coefficient. 2 RH-OCT-A images and 2 histological images (inner and outer forearm) per subject (n = 25).


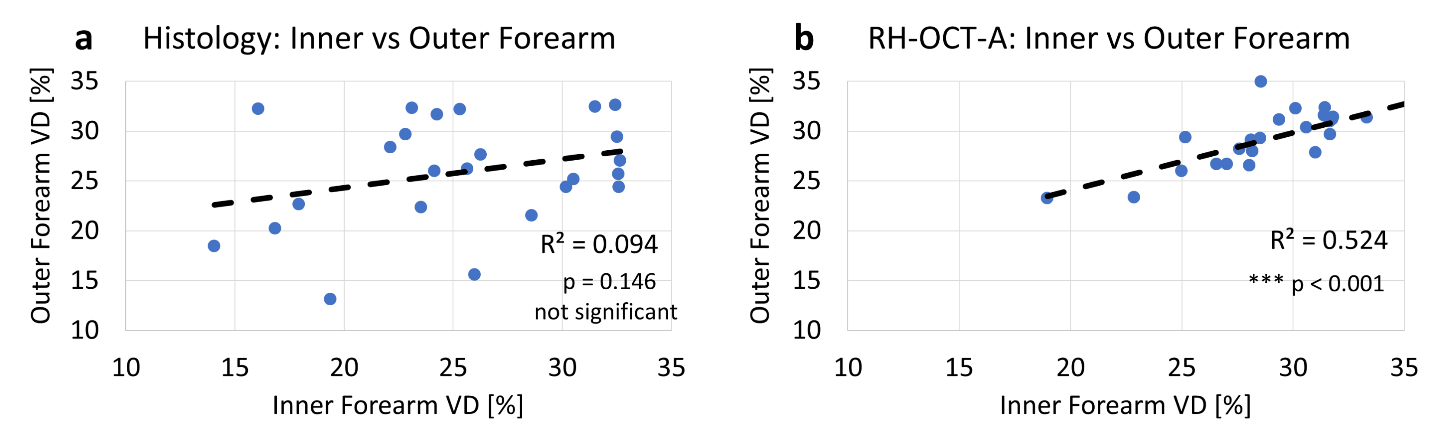


**Fig. S2.** Correlation between inner and outer forearm samples of the vessel density (VD) for histology and RH-OCT-A images for all 25 subjects. (**a**) There is no correlation of the VD for histology showing the variability between samples due to histological artifacts. (**b**) There is a significant (p < 0.001) correlation for RH-OCT-A images showing the reliability and consistency of this imaging technique. R^2^ is the square of the Pearson correlation coefficient.

| **Method** | **Cohort** | **Position** | **Vessel  Density [%]** | **Length of Vascular Network [mm]** | **Number of Branch Points** | **Vessel Diameter[µm]** |
| --- | --- | --- | --- | --- | --- | --- |
| OCT-A | Young | Ventral | 22.6 (± 1.7) | 7.2 (± 0.5) | 2300 (± 320) | 31.5 (± 3.1) |
|  |  | Dorsal | 22.8 (± 2.3) | 7.2 (± 0.7) | 2250 (± 350) | 32.0 (± 2.5) |
|  | Old | Ventral | 20.6 (± 3.2) | 6.8 (± 1.0) | 2255 (± 385) | 30.1 (± 2.0) |
|  |  | Dorsal | 21.6 (± 3.2) | 7.4 (± 0.9) | 2365 (± 370) | 29.1 (± 2.0) |
| RH-OCT-A | Young | Ventral | 30.7 (± 1.6) | 10.1 (± 0.6) | 3730(± 365) | 30.5 (± 1.7) |
|  |  | Dorsal | 30.7 (± 1.9) | 10.2 (± 0.8) | 3730 (± 540) | 30.3 (± 2.2) |
|  | Old | Ventral | 26.2 (± 4.0) | 9.0 (± 1.6) | 3080 (± 700) | 29.4 (± 2.1) |
|  |  | Dorsal | 26.7 (± 3.2) | 9.6 (± 1.3) | 3288 (± 670) | 27.8 (± 1.7) |
| Histology | Young | Ventral | 27.6 (± 5.2) | 12.5 (± 1.7) | 6091 (± 1032) | 22.1 (± 3.0) |
|  |  | Dorsal | 27.2 (± 4.9) | 12.1 (± 1.7) | 5700 (± 1060) | 22.5 (± 2.9) |
|  | Old | Ventral | 23.9 (± 5.5) | 11.0 (± 2.1) | 4970 (± 1345) | 21.9 (± 2.6) |
|  |  | Dorsal | 25.9 (± 4.6) | 11.5 (± 1.7) | 5380 (± 1055) | 22.5 (± 2.7) |

Table S1. Effects of sun exposure on inner (ventral) and outer (dorsal) forearm vasculature assessed by OCT-A, RH-OCT-A, and histology. No significant changes between the inner and outer forearm microvasculature, neither within the young, nor old, cohort were found.

|  | | | | | | |
| --- | --- | --- | --- | --- | --- | --- |
| Patient # | **Age [Years]** | **SBP [mmHg]** | **DBP [mmHg]** | **Height [cm]** | **Weight [kg]** | **Skin Type** |
| 1 | 20 | 94 | 54 | 162 | 52 | 2 |
| 2 | 20 | 107 | 61 | 163 | 61 | 3 |
| 3 | 20 | 110 | 73 | 168 | 54 | 3 |
| 4 | 21 | 111 | 67 | 170 | 57 | 2 |
| 5 | 22 | 138 | 74 | 165 | 79 | 3 |
| 6 | 23 | 116 | 67 | 160 | 67 | 2 |
| 7 | 23 | 124 | 68 | 173 | 64 | 3 |
| 8 | 25 | 99 | 70 | 168 | 68 | 2 |
| 9 | 25 | 117 | 64 | 163 | 59 | 3 |
| 10 | 25 | 119 | 77 | 170 | 70 | 1 |
| 11 | 25 | 120 | 71 | 170 | 61 | 1 |
| 12 | 25 | 120 | 74 | 175 | 113 | 2 |
| 13 | 27 | 139 | 86 | 163 | 68 | 2 |
| Young: | **23 (2)** | **117 (13)** | **70 (8)** | **167 (5)** | **67 (16)** | **2 (1)** |
| 14 | 66 | 114 | 66 | 158 | 57 | 2 |
| 15 | 66 | 136 | 62 | 167 | 69 | 3 |
| 16 | 66 | 137 | 65 | 161 | 107 | 3 |
| 17 | 66 | 156 | 76 | 165 | 68 | 2 |
| 18 | 66 | 166 | 77 | 161 | 90 | 2 |
| 19 | 68 | 133 | 61 | 158 | 59 | 2 |
| 20 | 70 | 123 | 82 | 164 | 87 | 3 |
| 21 | 70 | 134 | 63 | 160 | 70 | 2 |
| 22 | 71 | 172 | 74 | 152 | 47 | 1 |
| 23 | 72 | 121 | 63 | 149 | 62 | 2 |
| 24 | 76 | 120 | 66 | 150 | 54 | 3 |
| 25 | 78 | 144 | 76 | 165 | 75 | 3 |
| Old: | **70 (4)** | **138 (19)** | **69 (7)** | **159 (6)** | **78 (32)** | **2 (1)** |
| All: | **45 (23)** | **127 (19)** | **70 (7)** | **163 (6)** | **69 (16)** | **2 (1)** |

| Table S2. Subject Information |
| --- |


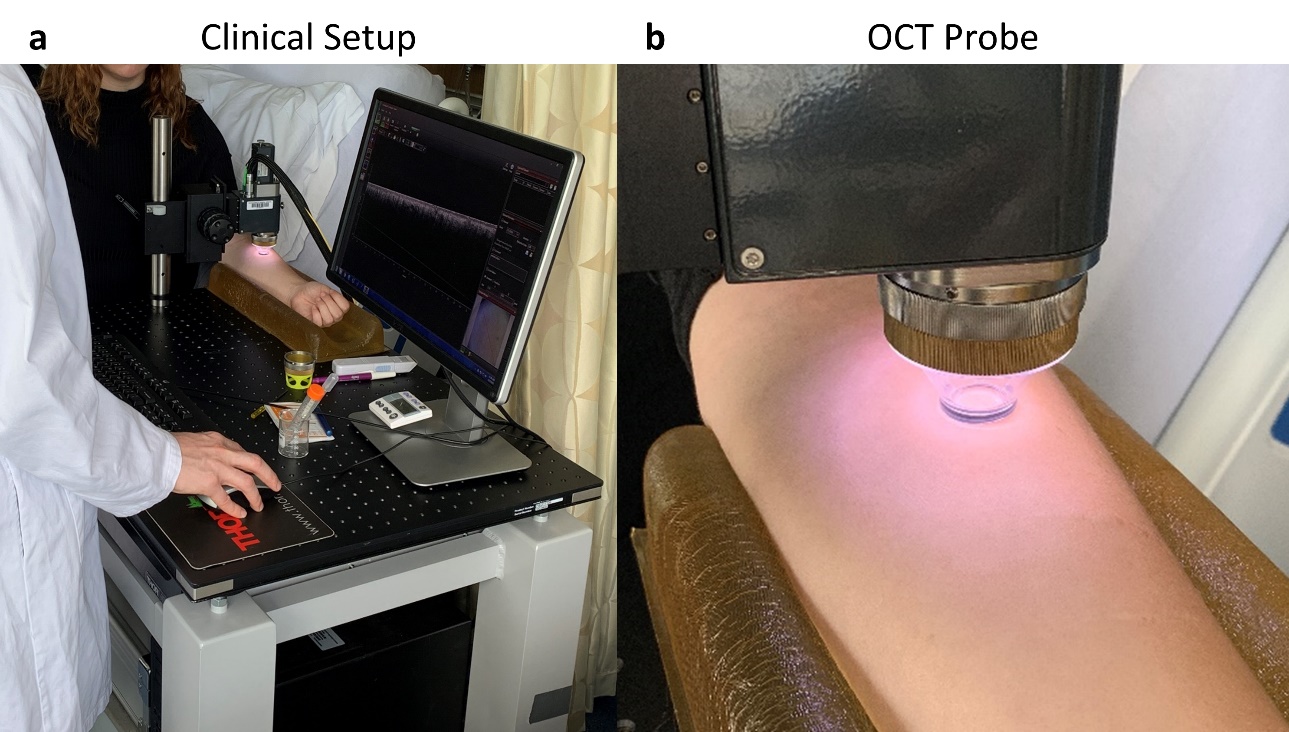


Fig. S3. Clinical setup of the commercially available OCT system. (**a**) The OCT system and all its components on a mobile cart. The subject is in a seated position with the forearm placed on a gel pillow. (**b**) The probe head is in contact with the skin for classical OCT-A, compression, and reactive hyperemia OCT-A images. An adjusting knob is used to move the probe head up and down.


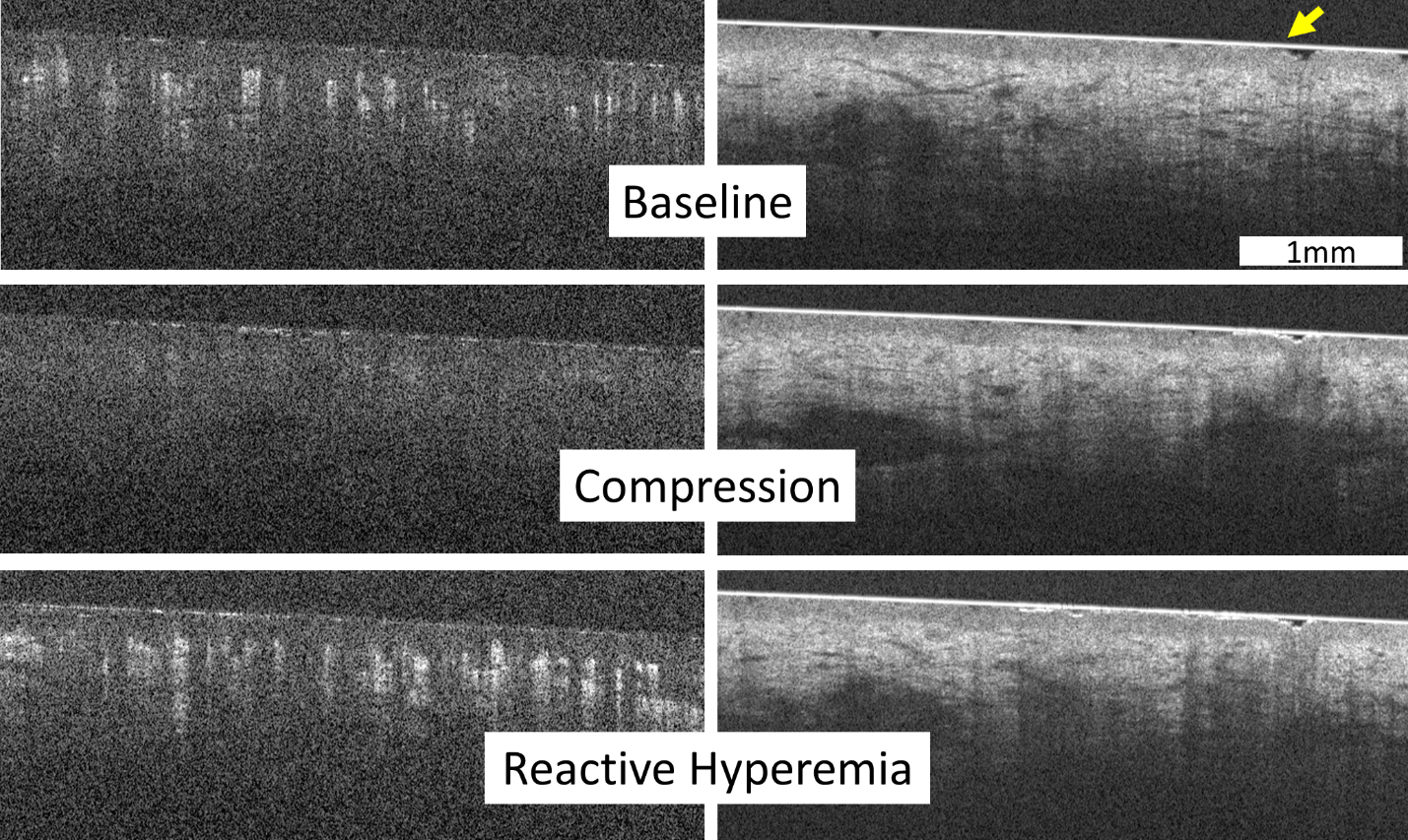


Fig. S4**.** Speckle variance (left) and morphological (right) baseline, compression, and reactive hyperemia OCT B-scans of a single plane of a representative subject. The yellow arrow denotes the glass spacer. The spacer is slightly angled to reduce reflection/interference artifacts. The tilt of the glass spacer was corrected before generating maximum intensity projections. The spacing is isotropic and the speckle variance B-scan is generated by using 2x slow axis averaging.


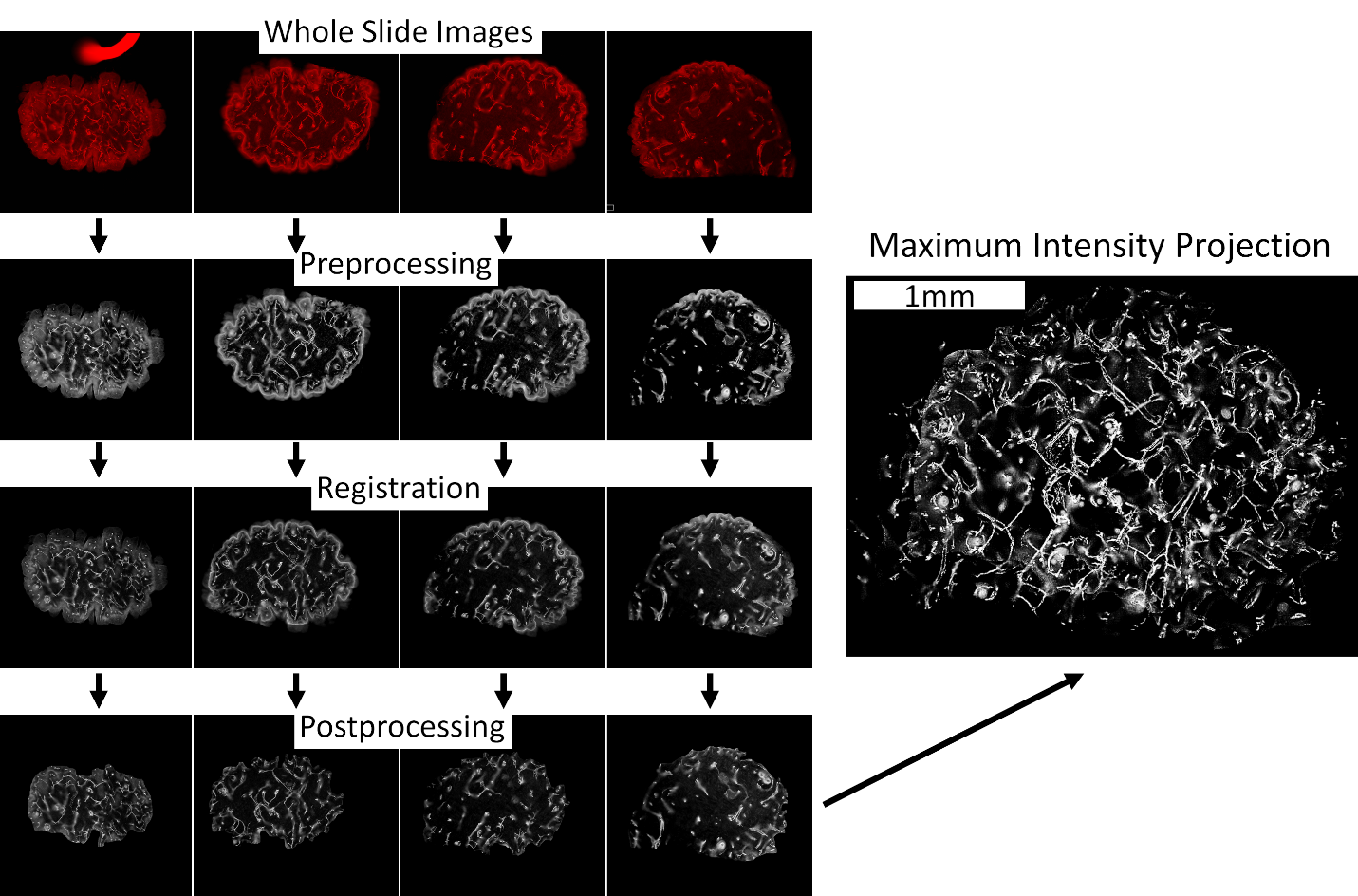
 Fig. S5. Schematic workflow showing the generation of maximum intensity projections (MIPs) of the microvascular network from 40 µm thick horizontal sections using a whole slide scanner.


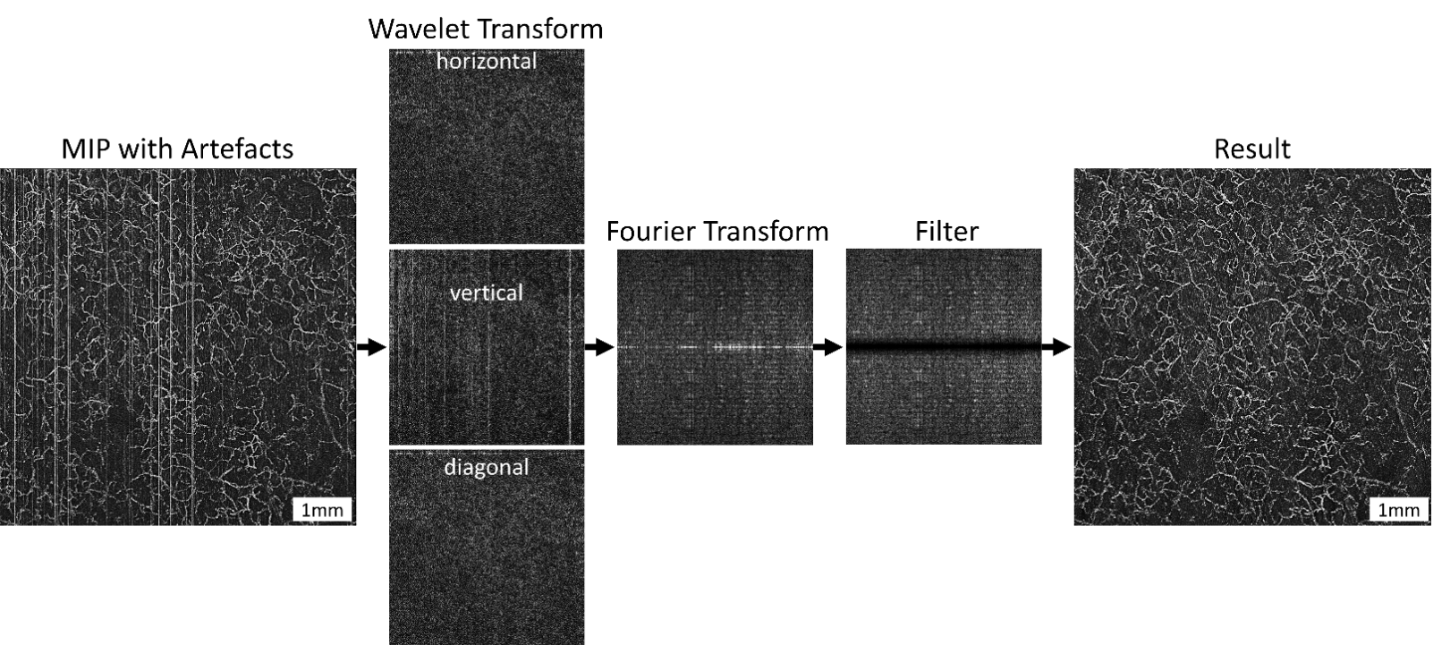
 Fig. S6. Movement artifacts, which manifested primarily as vertical stripes, were eliminated using a combined wavelet and Fourier filtering.
